# Resolvins as a Treatment Modality in Experimental Periodontitis: A Systematic Review of Preclinical Studies

**DOI:** 10.7759/cureus.21095

**Published:** 2022-01-10

**Authors:** Nouf Alshibani

**Affiliations:** 1 Department of Periodontics and Community Dentistry, College of Dentistry, King Saud University, Riyadh, SAU

**Keywords:** resolvins, periodontitis, periodontal therapy, eicosapentaenoic acid, docosahexaenoic acid, alveolar bone loss, animal models

## Abstract

This systematic review aimed to assess scientific data of existing literature to identify the efficacy of resolvins (Rv) in the treatment of periodontitis. The electronic databases, Web of Science (WOS), Medline/PubMed, The Cochrane Library, Scopus, and Saudi digital library (SDL), were searched for eligible studies in the field of periodontics. A thorough analysis of the retrieved literature provided five articles that were assessed and included in this systematic review. The quality of these studies was assessed by updated Essential Animal Research: Reporting of In-Vivo Experiments (ARRIVE) guidelines. The five included studies were published between 2005 and 2018 and investigated resolvins as a treatment approach in experimental periodontitis of animals. Among the study animals employed, New Zealand white rabbits were used in three studies, Wistar rats and Albino mice in two studies, respectively. Four studies have evaluated eicosapentaenoic acid-derived RvE1, and one study evaluated docosahexaenoic acid-derived RvD2. Oral-topical application of Rv was followed in four studies, and intra-peritoneal Rv injection was administered in one study. The study duration in these studies have ranged between 4-12 weeks, and the Rv dose was between 0.1 μg to 0.5 μg. One study evaluated the influence of RvE1 topical application on both the prevention and treatment of experimental periodontitis. Resolvins (RvE1 and RvD2) have been studied in periodontitis-induced animal models to assess their potential role in periodontal inflammation resolution. There are promising preclinical data of using resolvins as a treatment modality in experimental periodontitis. Resolvins have been demonstrated to inhibit the destructive inflammatory process and alveolar bone loss in laboratory-induced periodontitis under controlled experimental conditions.

## Introduction and background

Periodontitis is one of the most prevalent oral diseases characterized by inflammation of the periodontal soft tissues and the progressive loss of periodontal ligament and alveolar bone [1]. The two key causative factors in the etiology of periodontitis are an inflammatory host response and imbalanced interaction of the oral microbiota [2]. Gingival inflammation, clinical attachment loss, alveolar bone loss as evident through radiographs, locations with deep probing depths, tooth mobility, bleeding on probing (BOP), and pathologic migration of affected tooth are common signs of periodontitis [3]. The host response mediates irreversible death of the afflicted periodontal tissues due to the ongoing inflammation caused by simultaneous stimulation by bacteria and their by-products. The migration of leukocytes characterizes the pathogenesis of periodontitis, capillary dilation, increased blood flow, and permeability of blood capillaries [4,5].

In most cases, innate immunity triggers an inflammatory response that eradicates microorganisms and restores tissue homeostasis [6, 7]. The chronic inflammatory process is caused by the failure of the innate immunity to remove inflammation and clear immune cells and is marked by a prolonged and persistent inflammatory response to external stimuli such as bacterial components. Furthermore, inflammatory mediators such as cytokines, chemokines, and prostaglandins enhance the inflammatory response by attracting additional leukocytes and platelets to the infection site during chronic inflammation [6].

Periodontal therapy aims to reduce infection and inflammation, applying a more comprehensive range of interventions in a gradual and steady process [8]. The first step marks the control of supragingival biofilm by the patient and the professional, as well as those recognized risk factors in the aetiopathogenesis of periodontal conditions [9, 10]. In periodontitis patients, the first step is a prerequisite before applying the second step, based on the elimination of subgingival biofilm and calculus, which is the basic method of periodontal therapy. If the goals of periodontal therapy have not been met with steps 1 and 2, periodontal therapy using step 3 must be applied. This is accomplished with repeated subgingival instrumentation or other periodontal surgical treatments are used [11-13].

Non-surgical periodontal therapy (NSPT) refers to subgingival mechanical instrumentation in the second stage of periodontal therapy. The gold-standard therapy for stage I-III periodontitis is standard NSPT, mainly accomplished through scaling and root planning (SRP) [8]. However, certain patients and/or sites may poorly respond to regular NSPT. This could be due to microbial factors which are unable to convert the imbalanced infectious process to a more homeostatic/commensal balance, possibly due to subgingival biofilm remnants in the periodontal pocket after SRP [14], and/or tissue influx by periodontopathic bacteria [15] or the preservation of a due inflammatory process despite subgingival debridement. Therefore, there is a continuous search for adjunctive therapies that can improve the outcomes of subgingival instrumentation while considering the patients' health, potential adverse effects, availability, and cost [10, 16-18].

In that contrast, lipid regulatory substances have been well-appreciated for their role as signaling molecules in the inflammatory process because of their small size and specific impact on regulating infection. Specialized pro-resolving lipid mediators (SPMs), including resolvins, lipoxins, protectins, maresins, annexins, and specific peptides, are the most important lipid-derived mediators [19, 20]. SMPs are endogenously biosynthesized chemical agents with a dual role of pro-resolving and anti-inflammatory. They are generated during acute inflammation through enzymatic compounds' cyclooxygenase and lipoxygenase (LOX) pathways [19]. They have distinct paradigmatic properties and impede neutrophil activation, motility, and recruitment, limiting infection and altering neutrophil longevity. SPMs and their regulating cytokines can also help to regulate the adaptive immune system, reduce inflammation, and restore tissue homeostasis [21, 22]. These immunoresolvents differ from immunosuppressive molecules in that they not only reduce inflammation but also aid in the host defense [23-25].

Among the SPMs, resolvins (Rv) is of particular interest because of their exceptional anti-inflammatory and pro-resolution actions in various inflammatory disease conditions [25, 26]. Resolvins aid in the clearance of microorganisms and tissue regeneration in periodontitis and other infectious/inflammatory diseases [27-32]. The E-series resolvins (RvE) and D-series resolvins (RvD) are active molecules biosynthesized from the omega-3 fatty acids; eicosapentaenoic acid, and docosahexaenoic acid, respectively. In various animal models of inflammation, resolvins have demonstrated potent anti-inflammatory and pro-resolution effects [33-35].

There is insufficient evidence to support the clinical therapeutic efficacy of resolvins in the treatment of periodontitis. In recent years, however, the number of research evaluating the efficacy of resolvins in animal models has increased significantly. As a result, a comprehensive study of all preclinical data is necessary to establish their potential efficacy in humans. Therefore, this systematic review aimed to assess scientific data of existing literature to identify the efficacy of resolvins in the treatment of periodontitis.

## Review

Methodology

Study design and focus questions

The current systematic review was in accordance with the Preferred Reporting Items for Systematic Reviews and Meta-analyses (PRISMA) guidelines. The question in focus for this review was based on the PICOS strategy: "What is the role of resolvins in periodontitis of animal models with experimental periodontitis?"

The PICOS strategy is as follows:

Population: participants with a measure of periodontal disease; intervention: analyzing the role of resolvins; control (or comparator): participants with periodontal health; outcomes of interest: alveolar bone loss, a valuable clinical and radiographic parameter used in periodontology, especially for animal model studies [36].

The study design included in-vivo animal model studies.

Material Sources and Search Terms

The electronic databases Web of Science (WOS), Medline/PubMed, The Cochrane Library, Scopus, and Saudi digital library (SDL) were searched for eligible studies in the field of periodontics. Furthermore, a hand search of leading periodontology journals (Journal of Periodontology, Journal of Periodontal Research, Journal of Periodontal and Implant Science, and International Journal of Periodontics and Restorative Dentistry) was performed, followed by a grey search on Google scholar. The search was performed using accessible and Mesh English terms: 'pro-resolving agents,' OR 'pro-resolving lipid mediators in periodontitis,' OR 'periodontitis OR periodontal disease,' OR 'resolvins in periodontitis,' OR 'resolvins in periodontal inflammation,' OR ‘resolvins in periodontal resolution.’ The latest search was performed on October 15, 2021.

Study Selection and Data Extraction

The publications retrieved through database searches were imported into EndNote bibliographic reference management tool (v.x9, Clarivate Analytics, Thomson Reuters, Canada). The selection process was done in stages. Initially, the titles and abstracts were evaluated according to the eligibility criteria listed in Table 1. The articles included in the first stage were thoroughly analyzed and sorted according to full text by applying the same criteria in the next stage.

**Table 1:** Eligibility criteria applied

Inclusion criteria	Exclusion criteria	
Publications in the English language		Editorials, literature reviews, opinion articles, short communications, conference/poster proceedings
Studies evaluating the effect of Resolvins in experimental periodontal inflammation and/or the resolution in animal models		In-vitro studies
		Studies with no ethical approval
		Studies with no control group
Studies clearly state the experimental procedures, delivery methods, and evaluation duration.		Studies with inconclusive or inadequate data

The data from the included papers were extracted into pre-defined data extraction forms. The form includes the authors' names, year of study/publication, experimental design, study duration, outcome, and conclusion for each study.

Quality Assessment of the Included Studies

The quality of included studies was assessed by updated 2.0 Essential Animal Research: Reporting of In-Vivo Experiments (ARRIVE) guidelines containing 21 items checklist [37].

Results

Literature Search and Study Selection

**Figure 1:**
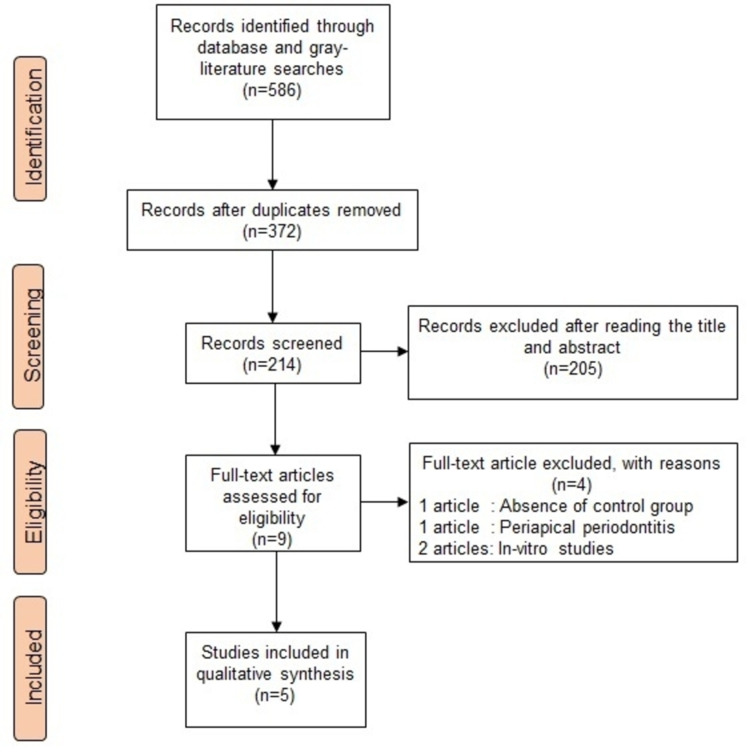
Literature search strategy

The literature search strategy followed in this review is presented in Figure 1. The initial search revealed 586 articles, in which 372 duplicated articles (obtained through different databases with the same title and author) were excluded. Another 205 articles were excluded after reading the title and abstract as they were not relevant to the topic being studied. The full-text screening of the remaining nine articles further eliminated five as they did not meet the eligibility criteria. Among the excluded studies, one study did not have a control group [38], one study evaluated Rv's effect in periapical periodontitis [27], and two were in-vitro studies [39, 40]. Finally, five studies were included in this systematic review [28-32].

Characteristics of the Included Studies

The five included studies were published between 2005 and 2018 and investigated resolvins as a treatment approach in experimental periodontitis of animals. Among the study animals employed, New Zealand white rabbits were used in three studies [28-30], Wistar rats [31], and BALB/c or B6 mice [32] in two studies, respectively. Experimental periodontitis was induced by a combination of ligature and oral gavage of *P. gingivalis* in three studies [28-30], ligature [31], and oral gavage of *P. gingivalis* [32]. Four studies [28-31] have evaluated eicosapentaenoic acid-derived RvE1, and one study evaluated docosahexaenoic acid-derived RvD2 [32]. Rv's oral-topical application was followed in four studies [28-31], and intra-peritoneal Rv was administered in one study [32]. The study duration in these studies has ranged between 4-12 weeks. The Rv dose used in these studies was between 0.1 μg to 0.5 μg. One study by Lee et al. [31], has evaluated the influence of RvE1 topical application on both prevention and treatment of experimental periodontitis (Table 2).

**Table 2:** Characteristics of the included studies

	Hasturk et al. (2006) [30]	Hasturk et al. (2007) [29]	Hasturk et al. (2015) [28]	Lee et al. (2016) [31]	Mizraji et al. (2018) [32]
Experimental animals	New Zealand white rabbits	New Zealand white rabbits	New Zealand white rabbits	Wistar rats	BALB/c or B6 mice
Experimental periodontitis	Ligature and *P. gingivalis*	Ligature and *P. gingivalis*	Ligature and *P. gingivalis*	Ligature	P. *gingivalis*
Resolvins/ placebo/control evaluated	RvE1 /ethanol/ systemic metronidazole	RvE1 /ethanol	RvE1/ethanol	RvE1/vehicle (not specified)	RvD2/ 2% carboxymethylcellulose (CMC) solution
Subject distribution	Experimental (n=6); Placebo (n=6); Systemic metronidazole (n=3); Ligature alone (n=3); No treatment (n=3)	Disease (n=5); Ethanol (n=10); RvE1 treatment (n=14)	No treatment (n=5); 4 μg RvE1‐treated (n=5); 0.4 μg RvE1‐treated; (n=5) Ethanol‐treated (n=5)	Prevention: No ligature (n=4); Ligature only (n=4); Ligature + RvE1 (n=4); Ligature + vehicle (n=4); Treatment : No ligature (n=6); Ligature only (n=6); Ligature + vehicle (n=3); Ligature+RvE1 (n=3)	Control; *P. gingivalis* +RvD2; *P. gingivalis*
Study duration	12 weeks	12 weeks	12 weeks	Prevention: 4 weeks; Treatment: 6 weeks	8 weeks
Resolvins application and concentration	Topical, 4 μg per tooth in ethanol on alternate days for six weeks	Topical, 4 μg per tooth in ethanol on alternate days for six weeks	Topical, 4 μg in 5% ethanol in saline/site or 0.4 μg in 5% ethanol in saline/site on alternate days for six weeks	Topical, 0.1 μg/μl on alternate days for four weeks in prevention group 4 ml of a 0.25 mg/ml solution on alternate days for six weeks in the treatment group	Intraperitoneally, Three doses of 0.5 μg RvD2 in 150 μl of the sterile saline solution followed by six doses of 0.1 μg of RvD2 over the next two weeks
Periodontal parameters evaluated	Alveolar bone loss	Alveolar bone loss + pocket depth + infrabony pocket depth + tooth mobility	Alveolar bone loss + pocket depth + infrabony pocket depth + tooth mobility	Alveolar bone loss	Alveolar bone loss
Outcome	Compared to other groups, topical RvE1 therapy at the ligature site reduced periodontal tissue and bone damage by >95%.	RvE1 treatment showed a statistically significant decrease in pocket depth and infrabony defect depth compared to baseline periodontitis and all other treatment groups (p 0.05). In contrast, those treated with vehicle alone resulted in 13% bone loss.	RvE1 (0.4 g/site) applied orally and topically showed a dose/response reduction of periodontal deterioration. RvE1-treated samples (4 g/site) showed good histological architecture and intact bone.	Prevention: When compared to control, RvE1 at low (0.1 g/l) and high (0.5 g/l) doses reduced alveolar bone loss by 1.51 and 1.73 mm^2^ (30%–40%), respectively. Treatment: The bone loss was reduced by 30–40% in the RvE1 group compared to the control.	The *P. gingivalis*-infected group had significantly lower residual bone volume compared to the other groups.
Conclusion	In rabbits with periodontitis, topical treatment of RvE1 provided significant protection against inflammation-induced tissue and bone loss.	RvE1 modifies the inflammatory response, causing it to resolve more quickly and effectively prevent the chronic phase. Tissue regeneration is aided by the removal of inflammation in the healing lesion.	RvE1, when applied as a topical nanotherapeutic drug, reduced atherosclerotic alterations and periodontal bone loss caused by periodontitis in an animal model of periodontitis and atherogenesis.	In the rat ligature-induced periodontitis paradigm, prophylactic RvE1 therapy dramatically reduces alveolar bone loss.	RvD2 therapy inhibits destructive inflammation and alveolar bone loss in mice with experimental periodontitis.

Primary Outcomes of Included Studies

Alveolar bone loss, a valuable clinical and radiographic parameter used in periodontology, especially for animal model studies, was evaluated in all the included studies [36], in addition to pocket depth and infrabony pocket depth in two studies [28, 29].

Quality Assessment Outcome

The quality assessment of the included studies was assessed by ARRIVE guidelines containing a 21-item checklist (Table 3), and the scores ranged from 16 to 18. No information was found on blinding or inclusion and exclusion criteria in any included studies. The ethical statement was missing in four studies [28-31]. One study by Mizraji et al. did not include the sample sizes of the study groups. Contrarily, all the studies were supported by strong background, experimental procedures, animal care and monitoring, outcomes measures, and study design [32].

**Table 3:** Quality assessment of the studies using ARRIVE guidelines

ARRIVE checklist items	Hastur et al. (2006) [30]	Hasturk et al. (2007) [29]	Hasturk et al. (2015) [28]	Lee et al. (2016) [31]	Mizraji et al. (2018) [32]
Abstract	1	1	1	1	1
Background	1	1	1	1	1
Objectives	1	1	1	1	1
Ethical statement	0	0	0	0	1
Experimental animals	1	1	1	1	1
Housing/Husbandry	1	1	1	1	1
Experimental procedures	1	1	1	1	1
Animal care and monitoring	1	1	1	1	1
Outcomes measures	1	1	1	1	1
Inclusion and exclusion criteria	0	0	0	0	0
Study design	1	1	1	1	1
Randomization	1	1	1	1	0
Blinding	0	0	0	0	0
Sample size	1	1	1	1	1
Statistical methods	1	1	1	1	1
Protocol registration	1	1	1	1	1
Results	1	1	1	1	1
Data access	0	0	1	1	1
Interpretation/scientific implications	1	1	1	1	1
Generalizability	1	1	1	1	1
Declaration of Interest	0	1	1	1	1
Scoring	16	17	18	18	18

Discussion

The current systematic review investigated the therapeutic effect of resolvins in experimental periodontitis of animals (mice, rats, and rabbits). Based on the findings of the included studies, resolvins have been identified as a host-modulator of the inflammatory response, causing it to resolve more rapidly and effectively prevent the chronic phase. Furthermore, by removing inflammation from the healing lesion, they inhibit destructive inflammation and alveolar bone loss, in addition to promoting tissue regeneration [28-32]. Their potential role in arresting the alveolar bone loss progression and alveolar bone regeneration makes them a potential therapeutic molecule in periodontal regenerative therapy [41].

Animal experiments are employed in various human health-related research domains, including drug development, biomedical research, experimental surgery, and environmental health. The use of periodontitis-induced animal models in a clinical trial provides further insight into possible therapeutic and/or preventive effects and interaction with certain physiological environments and supports decisions about human clinical research, such as whether or not to proceed to clinical trials [42]. Systematic reviews of animal studies are highly acknowledged for their relevance in identifying interventions with the best therapeutic potential for testing in clinical trials because they may provide robust and comprehensive descriptions of those animal studies. In making these decisions, the level of certainty in the evidence is crucial [39, 40]. In the present study, the ARRIVE recommended 21 items checklist was used to assess the quality and reliability of published research [37]. The 16-18 scores obtained in the quality assessment show that these studies were reliable and high-quality.

The combination of *P. gingivalis* and ligature model is commonly employed to induce experimental periodontitis in different animals, including mice, rats, dogs, and rabbits [43, 44]. Periodontitis induction modalities and duration are critical because they impact periodontal tissue loss and blood levels of proinflammatory cytokines, and different models can yield varied results. The ligature model has several drawbacks, including the trauma that occurs during ligature placement and disease severity decreases over time [44]. In contrast to the ligature insertion model, the oral gavage model does not cause acute alveolar bone loss [43, 44]. Ligature in combination with *P. gingivalis* infection results in more active periodontitis, increased periodontal bone resorption [45], and a higher systemic response than ligature or *P. gingivalis* infection alone and the disease intensity, being maintained over time [46]. Among the included studies, experimental periodontitis was induced by a combination of ligature and oral gavage of *P. gingivalis* in three studies [28-30], ligature [31], and oral gavage of *P. gingivalis* [32].

Periodontitis is marked by the host response's breakdown of connective tissue and bone. Inflammatory lipid mediators have a vital role in the aetiology of periodontitis. The exogenous PGE2 and LTB4, in particular, are significantly linked to the progression of the disease and are, in significant part, promoters of the chronic lesion [47, 48]. On the contrary, inflammation resolution is an active process, and homeostasis cannot be established until the lesion is clear of neutrophils [49]. These ideas are supported by recent investigations, which show that PGE2 and LTB4 augment the local inflammatory response, resulting in increased neutrophil recruitment and tissue damage caused by neutrophils [49]. RvE1 mediated inflammatory resolution reduces neutrophil infiltration, causes neutrophil apoptosis, and attracts nonphlogistic macrophages that phagocytize apoptotic neutrophils and microorganisms, clearing chronic inflammatory lesions by efferocytosis [19, 34]. During the clearance of inflammation, the release of proinflammatory cytokines and chemokines is reduced [19, 34]. Resolvins increase the resolution of inflammation in a feedforward, receptor agonist-driven manner, rather than inhibiting as in nonsteroidal anti-inflammatory drugs (NSAID) COX suppression or receptor antagonists [31].

Alveolar bone loss, a valuable clinical and radiographic periodontal parameter especially for animal model studies [36], was the primary outcome parameter of this review. The rabbit [28, 30, 37], rat [31], and mice [32] preclinical models were found to prevent experimentally induced alveolar bone loss. The regeneration of alveolar bone loss was demonstrated in the preclinical models in the rabbit [28, 29] and rat [31] models. The inflammatory responses triggered by bacteria are the primary cause of bone loss in periodontitis. Because of their antibacterial properties, antibiotics can help to prevent bone loss. However, antibiotics alone do not result in bone regeneration [30, 41], and in those situations, the role of resolvins is deemed significant.

Reduced osteoclast activity after RvE1 treatment appears to significantly regulate alveolar bone loss in animal models [31]. RvE1 reduces osteoclast differentiation in-vitro [50] and enhances osteoblast production in-vivo [51], according to previous studies. Furthermore, because nonphlogistic macrophages are required for tissue regeneration, RvE1 therapy-induced bone regeneration is expected to involve increased recruitment of nonphlogistic macrophages in the resolution phase [31]. Concerning RvD2, it has been shown that RvD2 treatment prevents alveolar bone loss by suppressing RANKL-mediated osteoclast development via osteoblast and T-cell signalling [32]. It has been proposed that RANKL (a transmembrane protein expressed on the osteoblasts surface) expression on CD4+ T-cells can directly impact the tightly controlled network of bone homeostasis and that osteoprotegerin (OPG) is a critical regulator of osteoclast development, activation, and survival [52, 53]. In experimental periodontitis, *P. gingivalis* is found to impact bone loss through modulating the RANKL-OPG ratio, and RvD2 therapy reduced the RANKL/OPG ratio [32].

Systemic conditions, such as cardiovascular disease, carotid artery disease, and cerebrovascular disease, have been linked to chronic periodontitis [54-56]. Patients with chronic periodontitis are more likely to have oral bacteria and endotoxins enter the bloodstream, stimulating a pro-atherogenic response in endothelial cells, resulting in systemic inflammation and fibrin deposition. With time, the elevated systemic inflammatory reaction leads to the onset and progression of atherosclerosis and cerebrovascular disease [57]. Although debatable, this association was further evident in the systematic review by Dietrich et al. [58]. In Hasturk et al. (2015) study, topical application of RvE1 reduced atherosclerotic alterations and periodontal bone loss caused by periodontitis in rabbit animal models of experimental periodontitis and atherogenesis. In the absence of oral lesions, topically applied RvE1 to the gingiva protected cholesterol-fed rabbits from atherogenic alterations. The anti-inflammatory effects of RvE1 were confirmed by a significant drop in CRP levels in the blood [28].

## Conclusions

Resolvins (RvE1 and RvD2) have been studied in periodontitis-induced animal models to assess their potential role in periodontal inflammation- resolution. There are promising preclinical data of using resolvins as a treatment modality in experimental periodontitis. Resolvins have been demonstrated to inhibit the destructive inflammatory process and alveolar bone loss in laboratory-induced periodontitis under controlled experimental conditions. Furthermore, their potential role in arresting the alveolar bone loss progression and alveolar bone regeneration makes them a potential therapeutic molecule in regenerative therapy. The use of such experimental periodontitis animal models, however, may make it challenging to generalize the findings to human conditions. In the future, clinical trials are deemed necessary to determine the efficacy of resolvins (RvE1 and RvD2) in humans.
